# In-depth characterisation of the tumour microenvironment reveals HHV-8-dependent immune regulation in HIV-associated and classic Kaposi sarcoma

**DOI:** 10.1038/s41416-026-03536-5

**Published:** 2026-08-05

**Authors:** Claudia A. M. Fulgenzi, Alessia Dalla Pria, Yiran Zhao, Alberto Giovanni Leone, Justin Weir, Ignazio Puccio, Krista S. Tuohinto, Päivi M. Ojala, Mark Bower, David J. Pinato

**Affiliations:** 1 Department of Surgery and Cancer, Imperial College London, Hammersmith Hospital, London, UK; 2 National Centre for HIV Oncology, Chelsea Westminster Hospital, London, UK; 3 Section of Virology, Department of Infectious Disease, Imperial College London, London, UK; 4 Fondazione IRCCS Istituto Nazionale dei Tumori, Milan, Italy; 5 Department of Histopathology, Royal Marsden Hospital, London, UK; 6 Translational Cancer Medicine Research Program, Faculty of Medicine, University of Helsinki, Helsinki, Finland; 7 Department of Translational Medicine, Università del Piemonte Orientale, Novara, Italy

**Keywords:** Tumour immunology, Tumour immunology

## Abstract

**Background:**

Kaposi’s sarcoma (KS) is the most common malignancy occurring in people living with HIV (PLWH). The clinical course of KS in PLWH established on combined anti-retroviral therapy (cART) resembles that of classic KS.

**Objectives:**

To compare the clinical and biological characteristics of HIV-associated KS in patients with well-controlled viral infection (*N* = 21) and non-HIV-associated KS (*N* = 19).

**Methods:**

Clinical data were prospectively collected from patients treated at the National Centre for HIV malignancies at Chelsea & Westminster Hospital. Targeted transcriptomic analysis and multiplex immune-fluorescence were performed on archival tumour samples.

**Results:**

Clinical outcomes were comparable between groups. The tumour microenvironment (TME) of non-HIV-associated KS was characterised by transcriptional upregulation of pathways associated with adaptive and innate immunity and angiogenesis. The TME of HIV-associated cases was associated with lower infiltration of activated CD4 cells. In both cohorts, we found the expression of HHV-8 genes to positively correlate with activated CD4, CD8, NK and immune checkpoint gene expression.

**Conclusions:**

The KS TME in the presence of well-controlled HIV infection is characterised by a lower degree of inflammation and more pronounced epithelial to mesenchymal transition. We also identified intra-tumoral HHV-8 gene expression as a driver of TME composition.

## Introduction

Kaposi’s sarcoma (KS) is a mesenchymal tumour of possible lymphovascular origin that recognises Human Herpes Virus 8 (HHV-8) infection as the necessary but not sufficient pathogenic mechanism [1]. Uncontrolled HIV infection represents one of the most common causes of KS development and progression [2] and, despite the drop in its incidence after the introduction of combined-anti-retroviral therapy (cART), KS remains the most common malignancy in people living with HIV (PLWH).

The current taxonomic classification of KS recognises classic, iatrogenic, epidemic, endemic and KS in men who have sex with men as five independent classes based on disease epidemiology. Recent evidence from our group has emphasised that the degree of baseline immune suppression, independently of the cause, is the main factor influencing the clinical course of KS [3], and reducing the degree of immune suppression leads to KS remission.

For instance, while formally included in the ‘epidemic’ group, HIV-associated KS includes clinical entities occurring in the presence of well-controlled HIV, resembling more closely that of classic KS rather than that of AIDS-associated KS. Equally, iatrogenic KS often has a clinical course that mimics that of AIDS-associated KS rather than other forms of KS [3]. In the case of KS persistence or recurrence, or when the underlying immune suppression cannot be treated, chemotherapy is effectively used to treat KS [4]. However, while immunosuppression remains key for KS development and progression, little is known about the biologic and immunologic characteristics of KS occurring in the absence of clinically significant immune suppression.

The strong pathogenic reliance of KS upon defective adaptive immunity makes the use of immunotherapy particularly appealing in this malignancy.

So far, immune checkpoint inhibitors have shown promising efficacy in both classic and HIV-associated KS [5, 6], with an objective response rate reaching 87% for the combination of ipilimumab (anti-CTLA-4) plus nivolumab (anti-PD-1) in classic KS [7]. Similarly, immunomodulatory agents such as pomalidomide have shown anti-cancer activity in both HIV and non-HIV-associated cases [8]. A better understanding of the biologic mechanisms leading to KS in the absence of clinically significant immune suppressions remains pivotal to develop new treatment strategies.

In this study, we aimed to investigate the differences between HIV-associated KS occurring in the presence of well-controlled HIV infection and non-HIV-associated KS without clinically evident immune suppression, by comparing the clinical characteristics and the composition of the tumour microenvironment (TME) of two matched cohorts.

## Methods

### Patient population

The two study cohorts were selected from a prospectively maintained dataset including all patients treated at the National Centre for HIV malignancies at the Chelsea and Westminster Hospital. The study included 40 consecutive patients with histology-proven KS diagnosed from 01/2008 to 02/2015 and available tissue samples. Diagnostic tissue was retrieved from the pathology archive. Patients with non-controlled HIV, defined as viral load >200 copies/ml and CD4 count <200 cells/mm^3^, or other causes of immune suppression were excluded. In the non-HIV cohort, only classical cases were included. Data collection was approved by the National Centre for HIV malignancies, and tissue collection was approved by the Imperial College Healthcare Tissue Bank, which holds authority for the collection, storage and use of human tissue appropriately under a single Human Tissue Authority licence (#12275) and ethical approval (Wales REC 3 22/WA/0214, R180090). All methods were performed in accordance with the relevant guidelines and regulations. Informed consent was obtained from all participants, and no identifiable images have been published.

Baseline characteristics are reported descriptively as percentages (%) for categorical variables and median with interquartile ranges for continuous ones. Categorical variables were compared using the chi-squared test, and continuous variables using the Kruskal–Wallis test. Progression-free survival (PFS) was calculated as the time from last treatment (either local or systemic) commencement to death or evidence of KS progression, whichever came first. Patients not reporting progression at the time of data cut-off were censored at the time of last follow-up. Median PFS was estimated using the Kaplan–Meier (KM) method, whereas median follow-up times were estimated with the reverse KM method.

### Transcriptomic profiling

After quality check for the presence of residual tumour, 3 scrolls by 10 microns were cut from formalin-fixed paraffin-embedded (FFPE) tumour blocks. RNA was extracted from each sample using the AllPrep DNA/RNA FFPE Kit by Qiagen as per the manufacturer’s instructions. After RNA quantification using the Nanodrop profiler, we performed gene expression profiling using the NanoString nCounter Analysis System with the PanCancer IO 360 Panel, to which a custom CodeSet of 10 HHV-8 genes was added (Supplementary Tables 1 and 2).

### Gene expression data processing and normalisation

Details about gene expression analysis are reported in Supplementary Method 1.

### Immunofluorescence

Slides prepared from 12 FFPE tissue blocks were obtained and processed for both immunohistochemistry and multiplex immunofluorescence (mIF) following standard protocols for clinical trial samples developed by the ICR/RMH Integrated Pathology Unit. Antibody staining was carried out using the BOND RX 3.0 Fully Automated Slide Stainer (Leica Biosystems).

To explore changes in immune cell populations and viral protein expression, 2 multiplex panels comprising 6 biomarkers each plus DAPI were developed using the PhenoImager HT system (Akoya Biosciences). Details about the methods are reported in Supplementary Methods 2 and Supplementary Tables 3 and 4.

### Plasma HHV-8-DNA quantification

Plasma HHV-8 was recorded at the time of tissue biopsy. Plasma HHV-8 samples were analysed using a quantitative nested PCR approach, targeting the open reading frame-26 (minor capsid protein) gene of KSHV.

## Results

### Clinical characteristics of classic and HIV-associated KS cohorts

The whole clinical cohort included 40 patients. Among them, 19 had non-HIV-associated KS (classic), and 21 had HIV-associated KS. Among those with HIV-associated KS, all of them were established on cART by at least 3 months at the time of sample collection, had HIV-RNA <200 copies/ml and all had CD4 count >200 cells/mm^3^. The baseline characteristics of the whole cohort and the two main subgroups are summarised in Table 1. In brief, most of the patients were males (*N* = 37), 24 out of 40 had prior loco-regional therapies to treat KS, with excision being the most common (*N* = 17); 22 received at least one line of systemic therapy. A higher proportion of non-HIV-associated cases received loco-regional treatments (73.7 vs 43.5%, Chi-squared test, *p* = 0.049). The proportion of patients requiring at least one line of systemic therapy was not significantly different between HIV-associated and non-HIV-associated cases (62.5 vs 42.1%, Chi-squared test, *p* = 0.183). Among patients who received systemic therapy, doxorubicin-based formulations were used as first-line treatment in 14 out of 15 (93.3%) patients in the HIV_associated cohort, and 8 out of 8 patients in the non-HIV-associated cohort (100%). As expected, people with high-risk KS were more likely to receive systemic therapy compared to low-risk patients (93.3 vs 33.3%). The median time from first KS diagnosis (either clinical or histology proven) and the last histology confirming KS (referred to the tissue used for the translational analysis of this study) was 6.8 months (range: 0–21 years). In those requiring systemic therapy, all the samples were collected prior to the start of the last line of treatment. As reported in Table 1, patients with HIV-associated KS had a younger age at first diagnosis, higher peripheral blood CD8 count and lower HHV-8 levels. Other baseline characteristics were well-balanced across the 2 cohorts. After a median follow-up of 15.3 months (95% CI: 12.0–30.3), median PFS in the whole cohort was 17.3 months (95% CI: 8.0–NA, Supplementary Fig. 1A), which was not different when stratified according to the HIV status (HR = 1.3; 95% CI: 0.50–3.48, *p* = 0.6, Supplementary Fig. 1B). Two patients (9.5%) in the HIV-associated cohort had a past medical history of multicentric Castleman’s disease (CD), which is another HHV-8 associated condition. In one of them, it occurred 12 years before the histological diagnosis of KS, and in the second one, it occurred 4 years before the KS histology. In both of them, there was no clinical evidence of active CD at the time of KS diagnosis.

**Table 1:** Baseline characteristics of the patient cohorts.

	Whole cohort (19) (*N*/%)	HIV-associated (21) cohort (*N*/%)	Classic cohort^a^ (*N*/%) (19)	Difference (*p* value)
Age at first KS diagnosis Median (IQR)	54 (42–67)	46 (36–59)	63 (52–73)	0.03
ECOG PS (0–≥1)	31–9 (77%–23%)	17–4 (80%–20%)	14–5 (74%–26%)	NS
Sex (M–F)	37–3 (92.5%–7.5%)	20–1 (95%–5%)	17–2 (89.5%–10.5%)	NS
Risk (0–1)	26–14 (65%–35%)	14–8 (66%–34%)	12–7 (63%–37%)	NS
Oral/Visceral involvement (Yes–No)	6–34 (15%–85%)	5–16 (24%–76%)	1–19 (5%–95%)	0.08
Number of loco-regional therapies Median (IQR)	1 (0–2)	1 (1–2)	1 (1–2)	NS
Number of lines of systemic treatment Median (IQR)	1 (0–2)	1 (0–2)	0 (0–1)	NS
HHV-8 level Median (IQR)	493 (0–1941)	100 (0–1521)	1190 (0–2140)	0.002
CD4 count (cells/mm^3^) Median (IQR)	641 (486–958)	642 (486–965)	639 (547–946)	NS
CD8 count (cells/mm^3^) Median (IQR)	638 (439–965)	688 (501–1192)	480 (286–671)	0.03

^a^In the non-HIV cohort, only classical cases were included.

### Targeted transcriptomic profiling reveals divergent polarisation of the tumour microenvironment in HIV-related versus classic KS

**Fig. 1:**
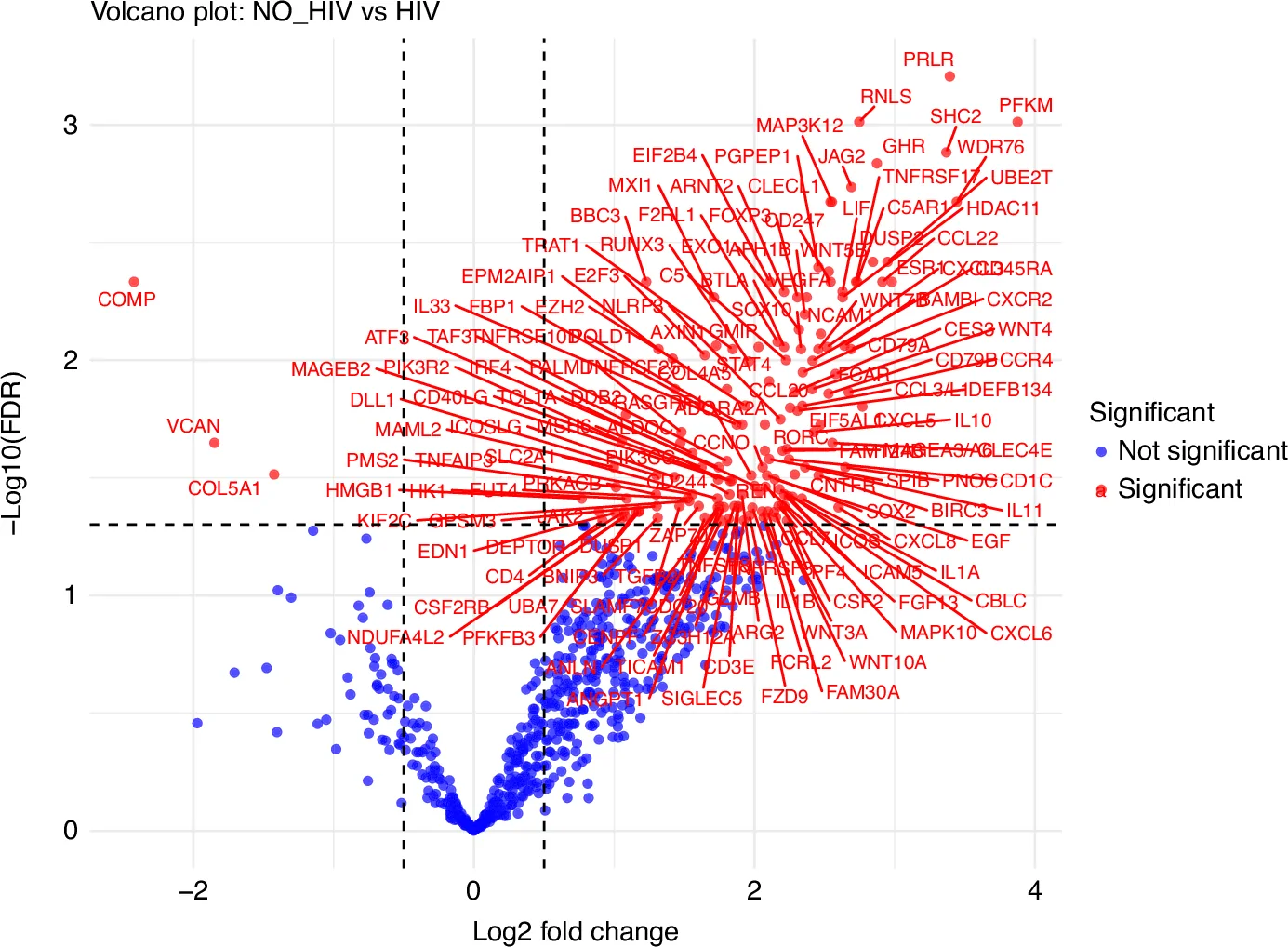
Non-HIV-associated KS is characterised by a different gene expression profile compared to HIV-associated cases. Volcano plot showing the differential gene expression between non-HIV cases (on the right) and HIV cases (on the left). Red genes represent significantly differentially expressed genes after FDR adjustment.

After removing samples with inadequate RNA quantity (*N* = 7) and those failing quality check (*N* = 8), 25 samples were retained for analysis: 13 in the HIV-associated cohort and 12 in the classic cohort. Baseline characteristics of the RNA evaluable sub-cohort are reported in Supplementary Table 9. Patients in the HIV-associated cohort were younger at the time of KS diagnosis and received more lines of systemic therapy compared to the non-HIV cohort. Differential gene set expression analysis showed significant upregulation of genes associated with innate and adaptive inflammatory responses in classic KS compared with HIV-associated cases (Fig. 1). Supplementary Table 5 reports the top 30 upregulated genes in the 2 cohorts. Pathway analysis demonstrated significant upregulation of gene transcriptional programmes related to allograft rejection, TNF-alpha, hypoxia, IL-6 and angiogenesis in classic KS, confirming enrichment of adaptive and innate immune pathways in classic cases (Supplementary Fig. 2A). Conversely, transcripts related to glycolysis and epithelial to mesenchymal transition were upregulated in HIV-associated cases (Supplementary Fig. 2B).

**Fig. 2:**
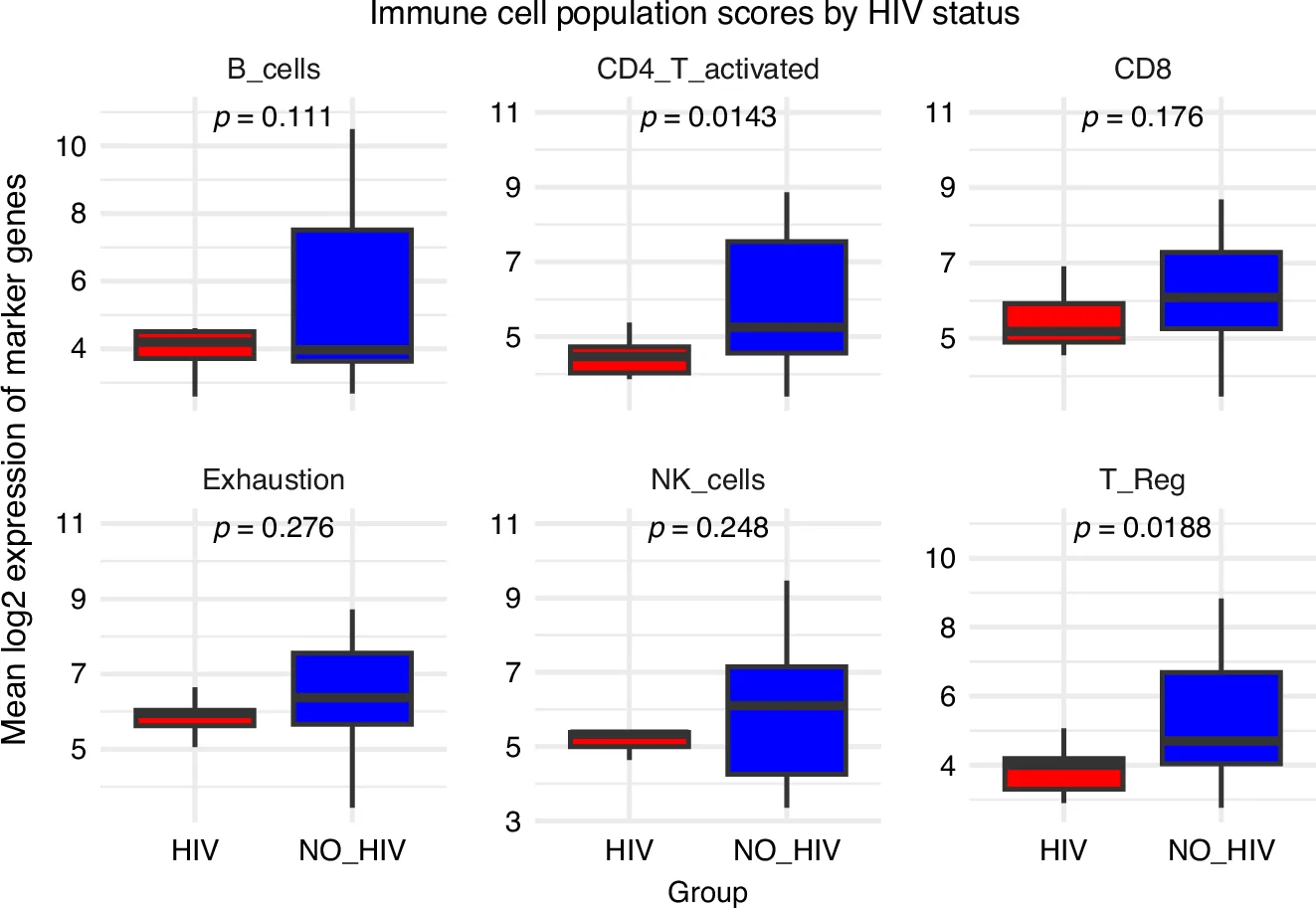
Non-HIV-associated KS is characterised by more pronounced immune infiltration compared to HIV-associated cases. Comparison of immune cells population detected by targeted transcriptomic in HIV-associated cases (red) and non-HIV cases (blue).

We then assessed the composition of the immune TME by calculating the immune score for six immune populations as reported in the ‘Methods’ section, and we observed higher representation of activated CD4 (*p* = 0.031) and regulatory T-cells (*p* = 0.019) in non-HIV cases, and a trend toward higher B-cell (*p* = 0.11) and CD8 infiltration (*p* = 0.18, Fig. 2).

### Differential expression of intra-tumoral HHV-8 genes according to the HIV status

Because we found that plasma HHV-8 DNA concentration was significantly higher in classic KS cases, we hypothesised that HHV-8 replicative activity might be differentially regulated depending on HIV status. To prove this hypothesis, we tested for the differential expression of a panel of selected HHV-8 genes according to HIV co-infection status. We found a significantly higher expression of HHV-8 K8.1 and v-IRF1 in classic KS, even though it showed only a trend toward significance after FDR adjustment (FDR = 0.085 and 0.124, respectively); only a modest correlation was observed between HHV-8 DNA levels and the intra-tumoral expression of selected HHV-8 genes (Supplementary Fig. 3). We then calculated the HHV-8 score for each sample, defined as the mean log2 expression of the viral genes, and dichotomised the population based on the median HHV-8 score into ‘high’ versus ‘low’. This confirmed a non-significant correlation between the HHV-8 score and plasma HHV-8 DNA. In the low HHV-8 group, the proportion of patients with HIV was numerically higher (66.7 vs 33.3%), and similarly, the high HHV-8 group had a numerically higher proportion of non-HIV samples (61.5 vs 38.5%), suggesting that higher HHV-8 gene expression may be required for the progression of KS in the absence of HIV.

### Intra-tumoral expression of HHV-8 genes is associated with a distinctive composition of the tumour microenvironment

Subsequently, as numerically different HHV-8 gene expression was present in the two cohorts; to define the impact of HHV-8 on the TME, we assessed the correlation between the expression of HHV-8 genes in the tissue and of the human genes included in the panel.

These revealed a positive correlation between the expression of the selected HHV-8 genes in the tumour and 202 host transcripts (Supplementary Fig. 4 and Supplementary Table 6), the ORA showed a positive association between the expression of viral genes and inflammation pathways, as well as pathways related to cells proliferation and epithelial to mesenchymal transition (Supplementary Fig. 7). As HHV-8 can exist in both latent and lytic phase in KS lesions, we assessed separately the expression of latent and lytic genes and their correlation TME transcripts. We observed that in the whole cohort, the mean expression of latent genes was higher compared to lytic ones (*p* = 0.03), in accordance with the latent phase being considered as the predominant state of infection. Whilst most of the upregulated TME genes correlating with viral expression overlapped between latent and lytic genes (*N* = 129), we identified a subset of genes that were exclusively associated with either lytic only (*N* = 73) (Supplementary Table 7), or latent only (*N* = 17) genes (Supplementary Table 8). The ORA pathway analysis showed that a stronger inflammatory response was associated with lytic genes, whereas genes significantly associated only with latent genes contributed to tumour-associated pathways; however, none of the pathways remained significant after FDR adjustment. This suggests a possible differential effect of latent and lytic genes in influencing KS pathogenesis and progression.

**Fig. 3:**
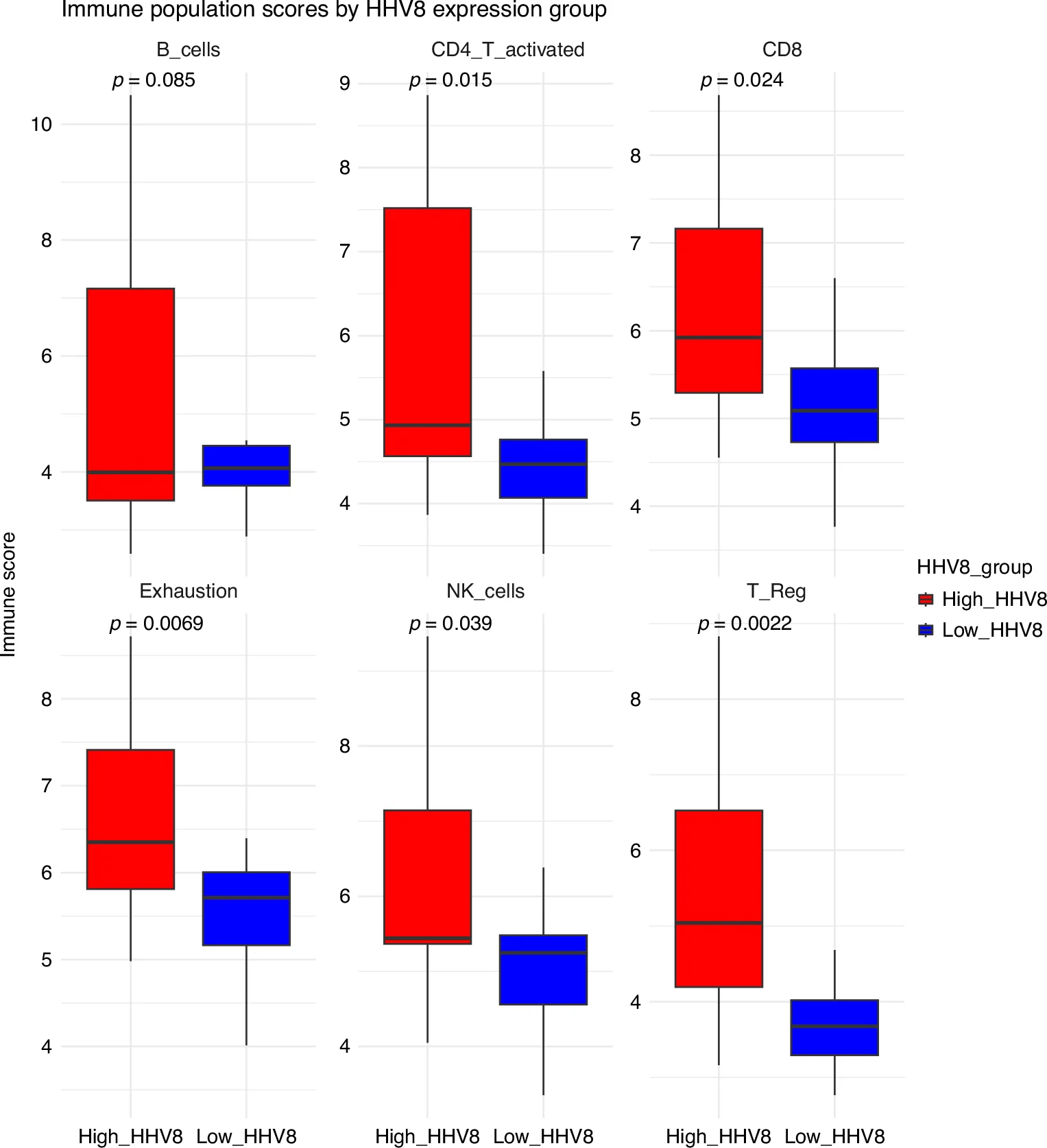
Higher expression of HHV8 genes is associated with more pronounced immune infiltration. Comparison of immune cells population detected by targeted transcriptomic based on the HHV8 score. “High” is reported in red and “low” in blue.

**Fig. 4:**
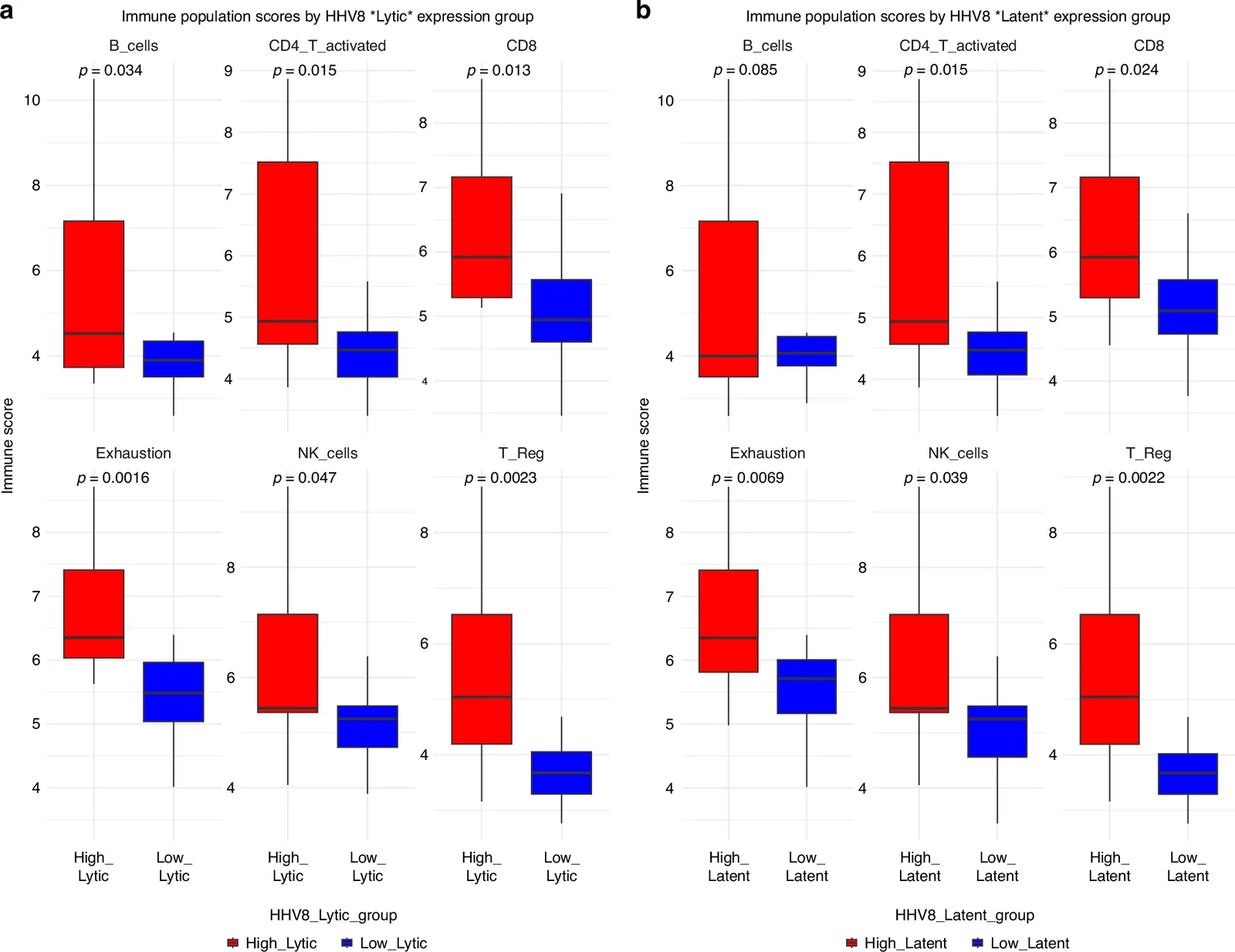
Higher expression of either lytic or latent HHV8 genes is associated with more pronounced immune infiltration. Comparison of immune cell population detected by targeted transcriptomic based on the Lytic (**a**) and Latent (**b**) HHV8 score. “High” is reported in red and “low” in blue.

We then compared the prevalence of each immune population, according to the HHV-8 score (Fig. 3). It showed that a high HHV-8 score was associated with higher expression of genes related to CD4-activated (*p* = 0.013), CD8-activated (*p* = 0.025) cells, NK (*p* = 0.039), T-regulatory cells (*p* = 0.002) and higher expression of gene sets associated with T-cell exhaustion (*p* = 0.007). The differences were maintained when calculating the HHV-8 score for latent and lytic genes (Fig. 4a, b) separately.

### Multiplex immunofluorescence highlights the differential distribution of T-cell subsets in HIV-associated KS

**Fig. 5:**
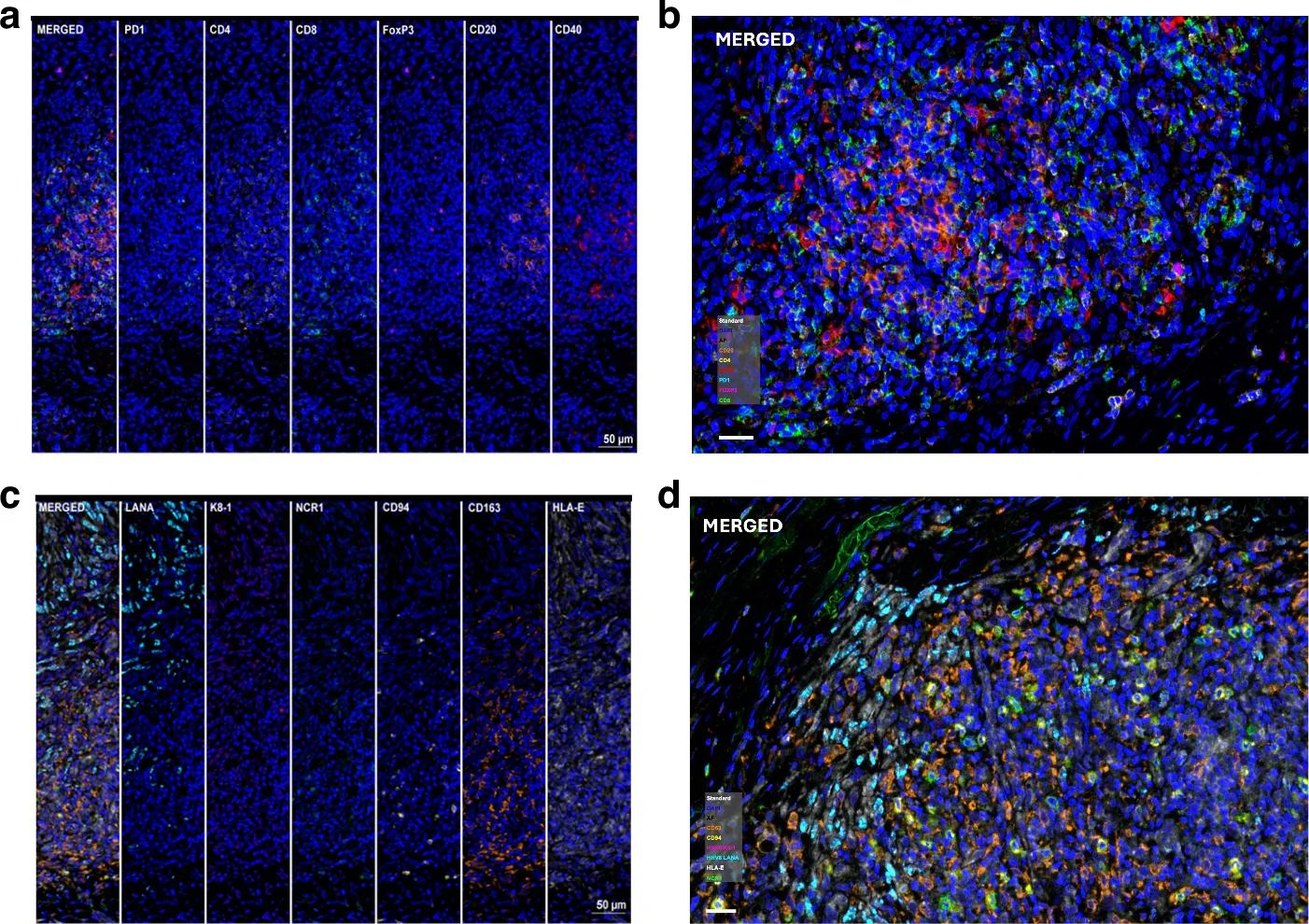
Multiplex immunofluorescence analysis of immune cell and HHV8-associated proteins. High-resolution image showing marker expression (Panel 1): Cyan, PD-1; yellow, CD4; green, CD8; purple, FoxP3; orange, CD20; red, CD40; blue, DAPI (nuclei); At ×20 (**a**), scale bar at 50 μm, and ×40 (**b**), scale bar at 20 μm. **c**, **d** High-resolution image showing marker expression (Panel 2): Cyan, LANA; purple, K8.1; green, NCR1; yellow, CD94; orange, CD163; white, HLA-E; blue, DAPI (nuclei). At ×20 (**c**), scale bar at 50 μm, and ×40 (**d**), scale bar at 20 μm.

To validate the RNA sequencing data, we performed immunofluorescence (IF) on 12 samples that had available material for both immunofluorescence and RNA analysis (HIV = 3; non-HIV = 9). The baseline characteristics of the IF sub-cohort did not differ between the two groups (Supplementary Table 10). Based on the biological relevance and adjusted *p* value derived from the differential gene expression experiments across the two cohorts (Table 2), 12 protein markers of interest were selected to validate immune cell distribution across clinical groups (Supplementary Tables 3 and 4, Fig. 5a–d).

**Table 2:** Target of antibodies used in the immunofluorescence panel, along with the adjusted *p* value of the differential gene expression (HIV vs non-HIV).

Immunofluorescence marker	Adj. *P* value DEG
FOXP3	0.005
CD4	0.05
CD20 (*MS4A1*)	0.32
PD-1 (*PDCD1*)	0.22
CD40	0.45
CD8	0.89
NCR1	0.26
CD94 (*KLRD1*)	0.35
CD163	0.40
HLA-E	0.86
HHV-8 K8.1	0.09
HHV-8 LANA	0.20

**Fig. 6:**
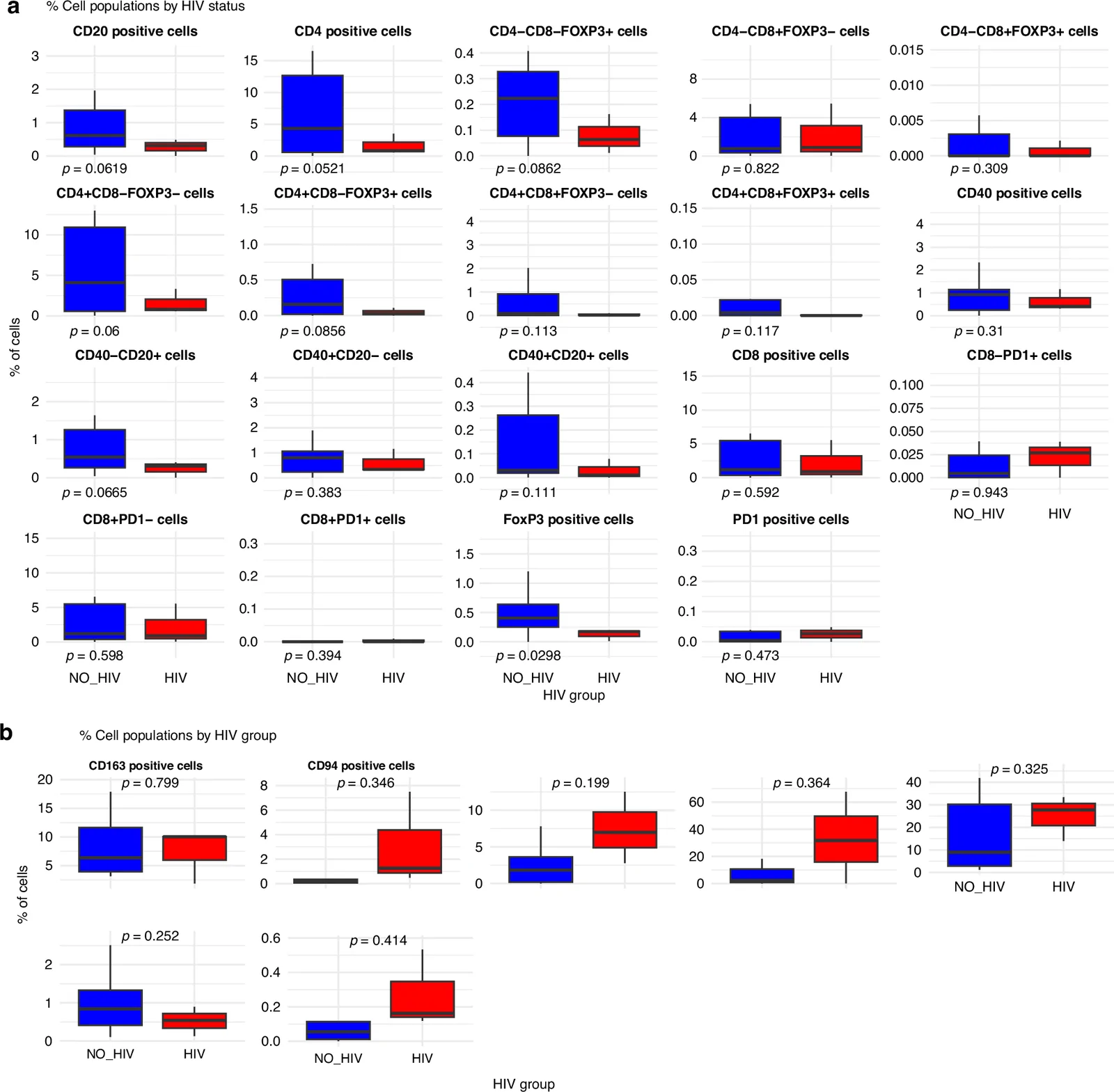
Comparison of immune cell populations by HIV status. Comparison of the immune cell population detected by immunofluorescence based on the HIV status. No-HIV is reported in blue and HIV associated is in red, in Panel 1 (**a**), and Panel 2 **b**).

As reported in Fig. 6a, b, we observed a higher proportion of CD4+ T-cells (*p* = 0.05) and regulatory T-cells expressing FOXP3 (*p* = 0.03) in the classic KS cohort, as well as a trend toward higher CD20+ B cells infiltration (*p* = 0.06), confirming targeted transcriptomics findings. Similarly, no difference in the other cell subtypes was observed.

**Fig. 7:**
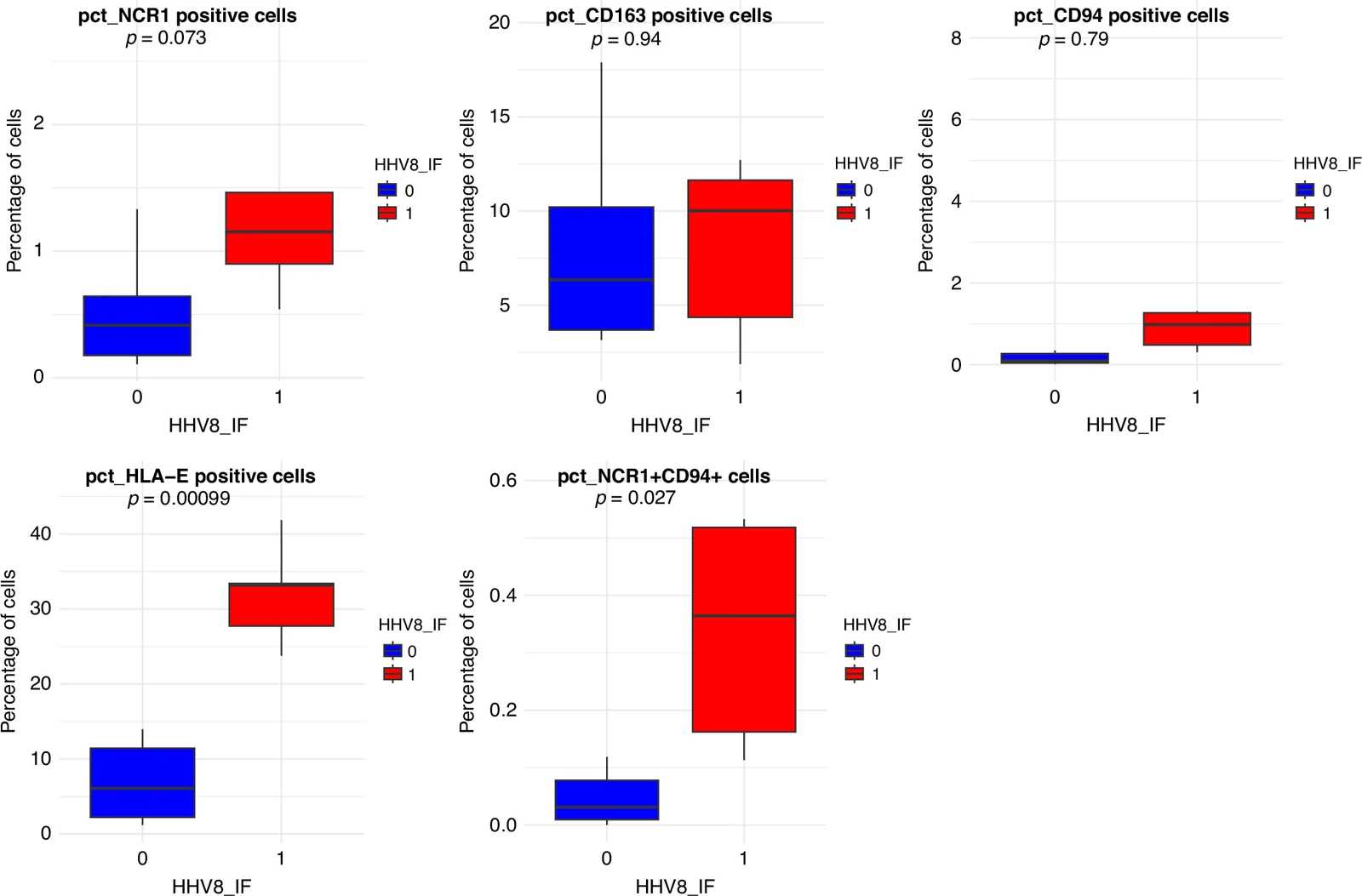
Comparison of immune cells population detected by immunofluorescence based on the expression of LANA and K8.1 in immune-fluorescence in Panel 1. Low expression is reported in blue, high expression is reported in red.

To assess the impact of HHV-8 expression on the immune microenvironment at the protein level, the IF cohort was stratified according to the percentage of LANA and K8.1-positive cells. The median percentage across all samples was calculated, and cases were classified as ‘high’ or ‘low’ based on whether their value was above or below the median. Samples classified as ‘high’ for both LANA and K8.1 were defined as having ‘high HHV-8 expression’, the remaining samples were classified as ‘low HHV-8 expression’. The proportions of immune cells, defined by markers included in the same IF panel as LANA and K8.1, were compared between the two groups using Welch’s *t*-test. In total, five samples were categorised as ‘high HHV-8’ and seven as ‘low HHV-8’. As shown in Fig. 7, ‘High HHV-8’ was associated with a significantly higher proportion of HLA-E positive cells (*p* = 0.001), a trend toward a higher proportion of NCR1+ NK cells (*p* = 0.07), and a higher proportion of NK cells expressing both NCR1 and CD94 (*p* = 0.027), which, upon binding with HLA-E, inhibits NK activity. These findings confirm a direct role of HHV-8 in harnessing the host immune response.

We then assessed the correlation between peripheral immune cells and intra-tumoral cells subtypes. As shown in Supplementary Fig. 6, we found a significant positive association between the peripheral CD8 count and CD4^+^:CD8^+^ ratio, and the different subsets of CD4^+^ and CD8^+^ subsets, including regulatory CD4^+^ cells, CD8^+^ and PD-1^+^CD8^+^ cells, whereas the peripheral CD4 count did not correlate with the explored intra-tumoral populations. This indicates a potential link between systemic inflammation and the composition of the TME in this subset.

## Discussion

HHV-8 infection is the unifying mechanism underlying the pathogenesis of the distinct classes of KS. By infecting blood and lymphatic vascular endothelial cells, HHV-8 directly contributes to tumorigenesis by promoting vascular proliferation, cell survival and apoptosis inhibition [9]. Alterations of adaptive immunity are fundamental pathogenic mechanisms that influence its development and progression. However, the functional characteristics of the KS tumour immune microenvironment occurring in the absence of clinically evident immune suppression are poorly defined, with detrimental implications for the development of novel therapies. In this study, we performed an in-depth characterisation of the TME of KS occurring in the absence of clinically significant immune suppression. Even if other studies have compared the TME of different subtypes of KS [10, 11], this is the first study focusing on KS occurring in patients without clinically evident immunosuppression.

By comparing two KS cohorts, as already reported in the literature, we found that PLWH were younger at first KS diagnosis, and had higher CD8 count [12]. The other baseline characteristics and the clinical outcomes did not differ between the 2 cohort. Furthermore, we found that no differences were found in PFS depending on the HIV status, confirming our previous findings that the degree of immune suppression is the main prognostic factor in KS [3]. Indeed, in this selected cohort of patients without clinically evident immune suppression, the PFS was not impacted by the HIV status by itself. Our translational analysis shows a more inflamed tumour immune microenvironment in classic KS compared to HIV-associated KS, despite comparable clinical characteristics. Our targeted RNA analysis using the Nanostring nCounter platform revealed an enrichment in inflammatory pathways, including TNF-alpha and IL-6 in classic KS, upregulation of pathways related to epithelial to mesenchymal transition and glycolysis was more common in HIV-associated KS. This was paired with higher expression of genes ascribable to activated CD4 and regulatory T-cells in classic cases.

These findings resonate with earlier evidence reported by Lidenge et al. in a comparative study, which was, however, confounded by the inclusion of AIDS-associated KS among the HIV-associated cases [13].

Furthermore, these results possibly underscore different pathogenetic mechanisms between the two subtypes, which point towards the requirement of a more profound inflammatory dysregulation for the development of KS in the absence of HIV. In particular, despite having a non completely understood effect in this context, FOXP3+ regulatory T-cells are thought to play a key role in KS pathogenesis, potentially by facilitating viral immune escape [14]. The higher proportion of FOXP3+ cells observed in non-HIV cases further supports the importance of the local immune microenvironment in the pathogenesis of classical KS. Although our bulk transcriptomic analysis does not allow us to distinguish between genes derived from tumour cells and those derived from immune cells, the higher expression of glycolysis-related genes in the HIV-associated cohort is of particular interest. Indeed, previous studies have reported that T-cells from PLWH exhibit higher rates of glycolysis, which are associated with more profound immune dysfunction. Similarly, cancer cells rely on this metabolic pathway, potentially competing with tumour-infiltrating immune cells for metabolic resources and thereby further contributing to immune escape in HIV-associated KS [15].

Using mIF, we sought to validate the gene expression results, while also assessing the proportion of other cardinal cell populations involved in anti-tumour immune control. Our findings confirmed higher infiltration of CD4 and regulatory T-cells in classic KS cases, while observing a relative uniformity of the other immune populations of interest across the two cohorts.

As HHV-8 is intimately linked to KS pathogenesis, we investigated how transcriptional activation of key HHV-8 genes relates to the composition of the TME across the two cohorts. While most of the assessed HHV-8 genes did not correlate with the peripheral HHV-8 DNA levels, a positive correlation was found between HHV-8 DNA level and intra-tumoral *ORF74* and *ORF75* - encoding two lytic proteins. Furthermore, a trend toward higher expression of *vIRF* and *K8.1* was observed in classic KS.

These results are provocative in suggesting the presence of a more pronounced viral lytic replication for KS pathogenesis in the absence of HIV. However, the limited sample size and lack of skin biopsies from non-tumoral sites constrain the interpretability of these findings. In our study, HHV-8 gene expression was positively associated with the overactivation of several inflammatory cellular pathways, including IL-6, TNFα, IFNγ and KS-specific pathways such as endothelial to mesenchymal transition [16–18] and angiogenesis independently from the presence of HIV. These results are consistent with the known complex interaction between HHV-8 and host immunity. HHV-8 is known to downregulate the adaptive immune response to avoid epitope-specific immune clearance. On the other hand, the virus can directly activate a pro-tumorigenic inflammatory response, which is in turn necessary for KS development [19].

In particular, it can activate the IL-6 pathway by its viral homologue [20], as well as inducing endothelial to mesenchymal transition and angiogenesis through, among others, *LANA* and *vFLIP* expression [21]. Despite being known to inhibit the host response, the production of IFN gamma and TNF-alpha is pivotal for KS development, and can be directly stimulated by viral proteins, such as OX2 [9]. Interestingly, we also observed a higher degree of T-cell infiltration and immune exhaustion in the presence of high HHV-8 latent or lytic transcriptional activity, independent of the HIV status. This highlights the central role of virus-host interactions as a tumourigenic mechanism in KS and a potential source of targeted treatment strategies in this oncological indication.

We acknowledge several limitations that might hamper the interpretation of our results. First, the retrospective nature of the study and the use of archival biological samples significantly limited the sample size. Second, the only partial overlap between RNA and immunofluorescence datasets restricted our power to validate the findings at the protein level. Third, the absence of multiple sampling from the same patients, as well as the lack of samples from normal skin or from patients affected by other KS subtypes, limits both the interpretation and generalisation of our data. Another limitation of our study is the lack of detailed characterisation of HHV-8 clades in the two cohorts. Given that different HHV-8 strains have been reported to be differentially distributed across KS subtypes [22], future studies are needed to determine whether specific clades are associated with distinct biological and immunological features. In addition, due to intrinsic limits of the selected methodologies and incomplete availability of clinical data, we did not investigate age‑related immune alterations, despite immunosenescence and metabolic comorbidities being recognised as permissive factors for classic KS [23, 24]. Despite these limitations, our study benefits from the use of two matched cohorts of KS occurring in the absence of immune suppression, providing a comprehensive comparative evaluation of two distinct clinical subtypes.

Overall, our data indicate that—despite similar clinical characteristics—a more pronounced activation of both adaptive and innate immunity pathways is evident in classic KS compared to HIV-associated KS occurring in the absence of immune suppression, despite an overall similar phenotypic composition of the tumour immune infiltrate. This difference may reflect distinct biological mechanisms that warrant dedicated preclinical investigation. We also highlighted the pivotal role of intra-tumoral HHV-8 gene transcription in shaping the local inflammatory microenvironment, providing a rationale to harness anti-viral immunity in the development of novel anti-KS strategies.

## Supplementary information

Supplementary

## Data Availability

Raw data will be made available upon request after the manuscript publication, pending approval by the corresponding author.
